# Observance to antiretroviral treatment in the rural region of the Democratic Republic of Congo: a cognitive dissonance

**DOI:** 10.11604/pamj.2018.31.159.15132

**Published:** 2018-11-02

**Authors:** Simon-Decap Mabakutuvangilanga Ntela, Nathalie Goutte, Jean-Manuel Morvillers, Cyril Crozet, Mathieu Ahouah, Marie-Claire Omanyondo-Ohambe, Bernard Ntoto-Kunzi, Félicien Tshimungu Kandolo, Monique Rothan-Tondeur

**Affiliations:** 1 Higher Institute of Medical Techniques of Kinshasa, Democratic Republic of Congo; 2 University of Paris 13, Sorbonne Paris Cite, Nursing Sciences Research chair, Laboratory Educations and Health Practices (LEPS), (EA 3412), UFR SMBH, F-93017, Bobigny, France; 3 University Hospitals of South Paris, Paul Brousse Hospital, INSERM 1193, DHU Hépatinov, France; 4 University Paris 13, Sorbonne Paris Cite, Laboratory Educations and Health Practices (LEPS), (EA 3412), UFR SMBH, F-93017, Bobigny, France; 5 Assistance Publique Hopitaux des paris (AP HP), Nursing Sciences Research chair Paris, France

**Keywords:** Antiretroviral therapy, cognitive dissonance, observance, rural region

## Abstract

**Introduction:**

This study aimed to understand the influence of local media, religion and cultural beliefs on the therapeutic compliance of patients living with HIV.

**Methods:**

This study was conducted in two rural hospitals in the Central Kongo province of the Democratic Republic of Congo. Semi-directional interviews were conducted with patients on antiretroviral therapy using a phenomenological qualitative method.

**Results:**

Our results indicated that patients living with HIV in the rural region of the Democratic Republic of Congo are in a constant state of tension between the messages for compliance to antiretroviral treatment advocated by caregivers, and those broadcasted by audiovisual media, religious leaders and local beliefs. This dissonance constitutes a real barrier to therapeutic compliance.

**Conclusion:**

Collaborative strategies between healthcare providers, patients, as well as religious, media and traditional organizations are urgently needed.

## Introduction

Human immunodeficiency virus (HIV) remains a major public health problem [1]. In December 2017, 36.7 million people across the world were living with HIV, among which 70% in Africa [2, 3]. Access to antiretroviral therapy (HAART) among HIV-positive individuals allows to reduce AIDS-related morbidity and mortality. In this respect, patient compliance is crucial for a successful treatment [4]. A good compliance to treatment may help to achieve the goal set fundamental by the United Nations Organization for AIDST, i.e. treating with antiretroviral therapy (ART) 90% of the patients and achieving a 90% viral suppression [5]. Nevertheless, subjects on antiretroviral therapy in rural areas of Africa, face various barriers that may undermine compliance to HIV-treatment [6]. Although communication approaches have been successful on HAART compliance behaviors [7], they also generated cognitive disruption [8]. In fact, after the diagnosis of the disease, the primary knowledge of HIV individuals about HAART is generally constructed on the basis of information provided by health workers, society, media and associations [9]. This information is quickly confronted and interpreted according to dynamic relationships between individuals [10] and may also be interpreted differently depending on the cultural context [11]. Several studies also showed that the whole system of information and communication around HIV raises major questions in Africa regarding potential disharmonies observed in diffusion channels [12]. Indeed, many shortcomings have been identified in operations concerning behavior change with regard to HIV prevention and compliance to treatment [12]. Jelliman *et al*. even argue that media are responsible for the stigmatization of HIV patients under HAART [13].

Our study was based on the well-known Festinger's theory on cognitive dissonance. This theory suggests that each individual has an inner desire to maintain all one's attitudes and beliefs in harmony [14]. However, this balance can be called into question when two or more cognitions come into opposition, generating a cognitive dissonance. This state is psychologically very disturbing for the individual [15], causing a state of tension which in turn motivates the need to recover a coherent cognitive universe [16]. As underlined above, compliance to therapeutic regimens is the result of a dynamic process of human behavior and interactions. Therefore, characterizing the quality of the information, the beliefs and how they relate to mental representations of patients may enable to identify possible barriers to compliance [17]. Several works have underlined the need to use good and appropriate information to properly deliver educational messages and to improve the effectiveness of follow-up [18]. Cognitive dissonance can exist when the situation experienced by an individual conflict with one's knowledge or beliefs. Vaidis used this theory to elucidate pre-existing attitudes and behaviors [19]. This approach was also used by Schoenfish to explain a risk that an individual can take in the when facing a well-known danger [20]. At Nsona-Nkulu Hospital in Mbanza-Ngungu and Saint Luc in Kisantu, patients are frequently confronted to regular local media speeches, traditional and religious beliefs concerning cure of the disease; however, the effects of these speeches on the observance of the patients receiving treatment are not well known. It seems therefore necessary to consider all cultural aspects in the promotion of compliance to HAART [21]. In this study, we aimed to understand the influence of the media, religious practices and traditional beliefs on compliance to HAART among patients from rural areas of the Democratic Republic of Congo.

## Methods

**Study sites:** this study was conducted in May and June 2016 in two rural hospitals in the central Kongo province (Nsona-nkulu of Mbanza-ngungu and Saint-Luc of Kisantu) in the Democratic Republic of Congo. We selected these hospitals for two main reasons. First, they are among the most important structures in rural areas treating such a large number of HIV-patients. Second, the authorities of these two sites expressed strong support for the conduct of the study.

**Study design and patients' recruitment:** we used a qualitative approach based on a phenomenological method in order to characterize patients' experiences and their own interpretations during the antiretroviral treatment process [22]. Adult volunteers (≥ 18), being on antiretroviral therapy for at least 3 months, speaking one of the three study languages (French, Lingala or Kikongo) were locally recruited. Consent was obtained through.

**Data collection:** semi-directional individual interviews were conducted in three languages, two local (Kikongo and Lingala) and one official (French). Open-ended questions were asked to patients using the language of their choice, allowing them to freely and easily talk about their disease and HIV-related issues [23]. All interviews were conducted in quiet locations, i.e. in HIV office of each health facility. In addition, interviews were carried out privately to ensure total confidentiality. Codes were used instead of names in order to obtain patients' consent to respect the anonymity of patients. Information was collected by a nursing researcher, specializing in public health and speaking the three before-mentioned languages. When patients refused to record their voice, information was written on paper, extending the time of the interview. The principal investigator was the only one involved in the process and also the only one to keep all the contents.

**Sample selection:** as this research focused on interviews accumulating multiple cases and based on oral histories with recorded and fully transcribed interviews, a contrast-saturation sample was preferred [24]. This saturation was reached when no new information enriched the study after a series of interviews as defined by Pires [25]. Indeed, Pires insists that one cannot go beyond 50 to 60 subjects because of the complexity it entails in the data analysis.

**Transcript:** the recorded information was faithfully and completely transcribed shortly after the interview on paper and on a computerized document in the language of expression of the patient by the researcher himself. After all the interviews, a summary sheet of each interview - mentioning the time, duration and place of the interview and the major lines of the context was filled in.

**Translation of interviews:** interviews recorded in local languages (Lingala and Kikongo) were translated into French by two professionals holding master degrees in these languages. Firstly, each expert carried out the translation independently. Secondly, a comparison of the translated texts was conducted after in order to detect the diverging points.

**Data analysis:** based on the transcriptions of each interview, a content analysis was performed using the Hsieh method [26]. This approach has been extensively used in nursing research studies [27]. This method enables to objectify the phenomenon in question. Codes with verbatim meaning were identified and then grouped into sub-themes. Then, a thematic and comparative analysis was carried out manually with the "sphinx Lexica" software. Codings expressing the aspects sought in the study were established. Finally, the verbatims producing dissonant effects (favorable and unfavorable) to the observance were removed.

**Ethical aspects:** the study was approved by the National Committee of Health Ethics (NCHE) of the Democratic Republic of Congo (decision N ° 017/CNES/BNPMMF/2016 of 08 January 2016). The authorities of each site selected for the study were informed before the actual start of the study and enabled to introduce us to head nurses and doctors in charge of the care of HIV-patients. An informed consent form written in French and translated into local languages (Lingala and Kikongo) was filled by the investigator for each patient. Thus, before beginning the interview, the interviewer read carefully the informed consent form in the language of choice of the patient. A copy of the consent form, was provided to interviewees, in case they have questions later. Providing an informed consent was a selection criterion for the enrolment in the study. Each interview was recorded using dictaphones and notes were also taken. Any patient for whom informed consent was originally sought was free to interrupt the interview and withdraw the consent at any time of the study. The confidentiality of data registration and notes was strictly respected. Written patient data were systematically stored in a locked cabinet. Computer files were protected using passwords. The recordings used during the analyses were destroyed afterwards.

## Results

A total of 50 interviews were conducted among patients aged between 19 and 79 years old, including 18 men and 32 women. The largest number of interviews (26) were conducted at the Nsona-Nkulu site (26 vs 24), including 43 conducted using the local languages (22 in Lingala and 21 in Kikongo). Interviews lasted on average less than 34 (±10) minutes.

**Disagreement in the media:** all patients confirmed to have received useful guidance and advices from healthcare providers, helping them to follow their HIV antiretroviral treatment. However, several participants revealed that anti-HAART propaganda was made through various messages broadcasted on local radio stations. These messages included claims for curative treatments for HIV, encouraging patients to stop HAART. "…at the very beginning of antiretroviral treatment, the nurses explanations helped me a lot and I was taking my medication appropriately. But…Last month I stopped taking my antiretrovirals for 5 days, especially because people are promoting certain products coming from India through local radios they do not like mixing with modern treatment, I had to interrupt the antiretrovirals temporarily and I took them back when I found that all they said was false." This conflicting information left patients in a state of permanent psychic tension, with doubts on whether or not taking antiretroviral treatment. "But above all we are confused. Should we go to those people who talk about the complete cure of AIDS through some local radios or should we continue with antiretroviral therapy …"

**Dissonance in the messages of religious leaders:** religions and religious leaders have an important role in the facilitation of the therapeutic compliance but may also be a barrier. Patients stressed that spiritual leaders of mother churches (Catholic, Protestant, Salvation Army…) often recommend patients to take into account caregivers' trustworthy advices. "… I was already discouraged, but my pastor convinced me to continue this treatment. When I saw him, he told me that he could pray for me, but his prayer would be effective if I continued with the treatment. Since then, I resumed treatment and I am better …" In contrast, other patients emphasized the negative role of charismatic churches called revivals, disorienting them by promising miracle cures related to their ability to castigate the evil spell. "In our church, the deacons initially told us that this disease was caused by sorcerers. They began by chasing away evil spirits for 4 months with towels, we were given holy water to drink instead of antiretrovirals "; "We went into exorcising by stopping the treatment for three months. Afterwards, I realized that on the contrary my health was getting worse … And since I restarted the treatment, my weight increased, I resumed certain activities …" Patients stressed that they were trying to listen to contradictory messages; on the one hand messages supporting antiretroviral therapy from the leaders of the traditional churches, and on the other hand messages from the revival churches advocating miraculous healing. In short, traditional churches urge the patients to start treatment as early as possible while in contrast revival churches recommend to delay the start of antiretroviral therapy because they associate mysticism to the disease, using exorcism to care it. "… ohhhh the priest of the parish prayed for me, he advised me to go back to hospital in order to continue the treatment after the prayer. My sister did not trust the priest and took me to their pastor. The latter rather said that my illness was from a mystical cause whose healing is only spiritual … I spent a long time without treatment, … I was confused "At the revival church, they told me not to start antiretroviral therapy because it was first necessary to get out the evil spirits that are in me … That's why I started my treatment very late… when I met the spiritual chief of our Kimbanguist church, he blessed me and asked me to go back to hospital and start my treatment as soon as possible. This supported me, and I decided to resume the treatment." All these contradictory messages from the spiritual leaders of mother and revival churches raise questions on the right path to follow among patients who seem really disoriented by the strategies adopted by revival. Generally, this is only after their health condition deteriorates that patients resume treatment. Thus, it would seem that health deterioration was the main factor that forced patients to start antiretroviral therapy. "I sometimes interrupted it, especially when religious care required it. But when I saw that my health was collapsing, I decided to break this path. And since I took my antiretroviral treatment, it´s fine now ".

**Figure 1:**
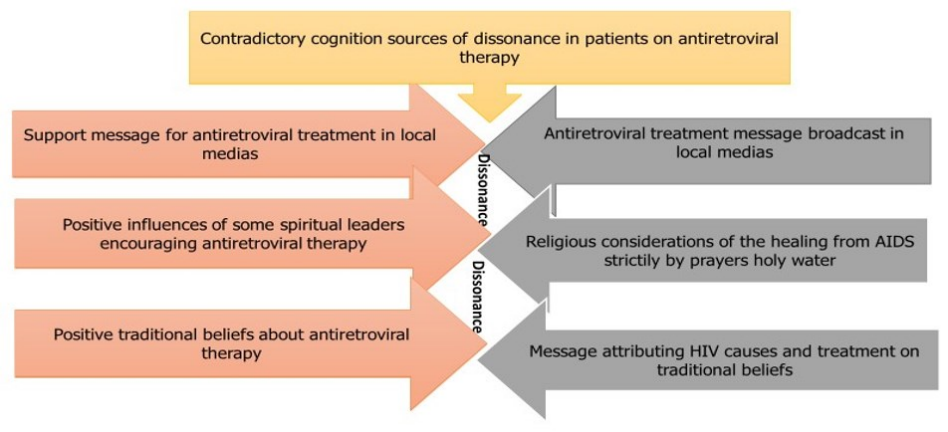
Different sources of dissonance

**Dissonance in traditional beliefs:** some patients also mentioned that in chronic diseases, cultural beliefs, traditions and customs still have a strong hold in spite of the social evolution. These cultural beliefs may raise doubts on the effectiveness of the antiretroviral treatment. "… In our country, we resort to modern medicine especially for acute disease. However, the path of traditions is not yet neglected. Since my childhood, this is the way that our parents used to cure us of any incurable disease … no, I doubt that antiretroviral treatment will really work…" Moreover, in most cases, patients are in conditions of vulnerability and feel victims or accused. That is why, very often, family members may take the lead in the management of their disease. "I cannot decide…My brother forced me to take traditional treatments. I was frustrated, stressed out…" Several patients also reported seeing the "modern" medical doctor during the antiretroviral treatment process but also at the same time traditional healers due to the insistence of their family. Patients listed the different strategies used by traditional healers to "cure" their disease among which intake of herbal teas or resort to tatoos " Many people advised us to go to hospital, to see a doctor or a nurse, at the same time we were advised to resort to tattoos, to consume several herbal teas we are wondering on whom to rely". Other patients indicated that the use of fetishism during antiretroviral therapy may be relevant. In most cases, these fetishists require VIH patients to drop antiretroviral therapy during the incantation period. However, patients generally resume treatment once their health has deteriorated. "I gave up on antiretroviral treatment because I had to adhere to the instructions of the fetishist doctors. Imagine that one of them made me drink the blood of a hardly slaughtered fowl. Another fetishist doctor made me sleep under the bed for 4 nights. While I spent time with the fetishist doctors, my health changed in a very negative way. I decided to go back to the antiretroviral treatment, otherwise it would be very bad for my health ". In Africa in general, HIV is often considered as a bad luck thrower disease. This design prevents people living with HIV from initiating antiretroviral therapy as soon as possible. Moreover, for patients on antiretroviral therapy, this cultural belief arouses the appearance of a cognitive dissonance Figure 1.

## Discussion

Our study enabled us to better understand the influence of media, religions and cultural beliefs on compliance to antiretroviral therapy in 50 patients from rural regions of RDC. The phenomenological based approach also permitted us to thoroughly investigate patients' personal experiences [24]. We adapted this approach in the context of patients receiving antiretroviral therapy in order to understand their reactions when facing different speeches from medias [25]. Participants in our study revealed the different sources of dissonance they faced.

**Dissonance in broadcast messages:** our findings indicate two opposite media views r regarding antiretroviral therapy: one supporting antiretroviral therapy and the other broadcasting anti HAART messages. This indicates a dangerous lack of control as regards broadcast messages. And yet "any communication act should be part of the cognitive environment of the population and should be understood on the basis of the experience of its realization [12]. Our findings regarding the disharmony on HIV/AIDS in press corroborate those of other studies [26]. For instance, in South Africa, President Thabo Mbeki´s has considered Africa as a victim of AIDS. This position in the media on HIV/AIDS caused confusion among the population [27]. In addition, mass media, especially newspapers and magazines, very often stigmatize population by only focusing on dramatic effects of the disease and by considering the epidemic as a major event [28]. Thus, "the myths on AIDS reflect a diversity of representations in Africa. This reinforces the urgent need to adapt messages according to the context of their dissemination" [29]. Inconsistencies and contradictory messages from communication channels prevents from a correct understanding of messages. They also, create cognitive conflicts responsible for behavioral changes [12]. An effective control of message broadcast concerning health issues seems therefore of high importance to enable a better compliance to treatment.

**Dissonance in the messages from religious leaders:** traditional African religion plays an important role in individual's day life, as an essential element of the "indigenous knowledge systems". However, this "knowledge" also appears to be an important source of dissonance among patients with VIH. Our results are in line with other studies carried out in rural regions showing that spirituality plays an important role in the self-regulation of the disease and its treatment [30]. Our results are also in accordance with a study by Gruénais on religious congregations [31]. In our survey, we showed that religions had both good and bad influences on AIDS treatment in the Democratic Republic of Congo. We observed in our study that mother churches (Catholic, Protestant, Salvationist, Kimbanguist …) and their spiritual leaders seem to a positive influence on compliance of HIV patients to treatment by encouraging them to take their anti-retroviral treatment. As stressed by Denis P [32] traditional African religions are key elements in the fight of AIDS. In contrast, revival churches seemed to have strong negative influences on HIV patients by promising miraculous cure. This can be related to the fact that for some patients AIDS is a divine punishment [33]. This trend has been criticized by the Congolese press, which showed the drift of numerous Congolese pastors who encourage HIV patients to stop the HAART to hope for a miracle cure [34]. We showed that most patients do trust their religious leaders. This trust toward religious leaders can be used to help convincing patients to better follow their treatment.

**Dissonance on traditional beliefs:** beliefs in tradition are the third major reason which creates psychic tension among patients with. Such beliefs on antiretroviral therapy (ARV) are crucial for the compliance with treatment but are still too poorly documented in sub-Saharan African countries [35]. Kemppainen *et al.* [36] demonstrated that the majority of participants did believe that HIV was a serious and chronic disease that can be controlled through appropriate antiretroviral therapy, although traditional treatments still remains, the main medication against diseases. In addition, it has also been showed that individuals who believed that the cause of HIV/AIDS was due to misfortune or God's will were also those who were more likely to believe that the progression of their illness was linked to destiny [30]. We also found that compliance to treatment was also related to patients' ties with their family and how they communicate with their relatives [36]. Traditional aspects have influenced the use of antiretroviral drugs from the beginning, which is mainly represented by the consultation of traditional healers in HIV care. Traditions have thus strong impacts on compliance to treatment as most of patients may have seen traditional healers immediately after the diagnosis of their disease. Limitations of the study: Subjects were volunteers and the study were limited to two hospitals. Caution is therefore needed when extrapolating the results.

## Conclusion

Our results suggested that the speeches from religious leaders or from media generate a major cognitive dissonance among patients with HIV. Patients find themselves torn between different beliefs, leading to a state constant tension, and causing non-compliance with treatment. Effective strategies to improve the collaboration between the different stakeholders (healthcare providers, patient's media and traditional organizations) are urgently needed. Considering all cultural and traditional aspects is also of high relevance in the fight against HIV. In addition, educational interventions and campaigns involving various stakeholders such as caregivers (nurses, doctors…), opinion leaders (religious leaders, media and customary chiefs) as well as local associations raising awareness of the importance of antiretroviral therapy should be implemented among rural populations. Other studies of this kind are needed in similar contexts.

### What is known about this topic

- Africa remains the continent most affected by HIV infection;
- The general prevalence of HIV in the Democratic Republic is low, but with the hidden epidemic in rural areas;
- Chronic diseases in the Democratic Republic of Congo are influenced by culture.

### What this study adds

- Some local media are making antiretroviral propaganda in the context of this study, which propaganda has never been denounced. Cognitive conflicts caused by discreet media in patients on antiretroviral therapy create dissonance that strengthen antiretroviral treatment non-compliance;
- Sensitization of opinion leaders to the control of the messages broadcasting by medias in connection with the patient's health;
- The mobilization and involvement of cultural leaders (some spiritual leaders, opinion leaders, etc.) as a strategy to improve adherence to antiretroviral treatment would be a good therapeutic education strategy in this context.

## Competing interests

The authors declare no competing interests.
